# Use of Micro Bioreactor systems to streamline cell line evaluation and upstream process development for monoclonal antibody production

**DOI:** 10.1186/1753-6561-5-S8-P14

**Published:** 2011-11-22

**Authors:** Steve R C Warr, Jai Patel, Rongzan Ho, Katy V Newell

**Affiliations:** 1 GlaxoSmithKline, Stevenage, Hertfordshire, SG1 2NY, UK

## Introduction

The development of monoclonal antibody (mAb) processes conventionally involves the generation of multiple cell lines in multiwell plates, cell line screening in shake flasks followed by final cell line selection and process optimisation in bioreactors. This process can typically take up to 18 months to generate a robust process suitable for early phase manufacturing and therefore any opportunity to streamline this process impacts directly on drug development timelines.

This work describes the potential integration of the Micro-24 Bioreactor system (Pall Life Sciences) and the Duetz Microflask system (Applikon Biotechnology) into cell line development and early process development and demonstrates how these systems can be used for cell line evaluation and process optimisation. The Micro-24 Bioreactor system comprises 24 bioreactors (7ml working volume) each capable of independent temperature, dissolved oxygen and pH control. Cell cultures are carried out in a presterilised polycarbonate mammalian cell culture cassette with a central vent and are inoculated manually in a laminar flow cabinet before sealing with Type A single use closures and incubation under experimental conditions.

## Methods

The performance of a number of cell lines in the Micro-24 Bioreactor and the Duetz Microflask system was compared to that in shake flasks and bioreactors using cell numbers, viability and product titre. This data was then used to rank cell lines according to specific parameters. Unless otherwise stated a standard hydrolysate containing complex medium and standard experimental conditions were used throughout this work. For shake flasks these were: 35°C, 5% CO_2_, 140 rpm; for Duetz Microflasks: 35°C, 5% CO_2_, 200 rpm, 80% humidity and for Micro-24 Bioreactors: 35°C, 650rpm, 6.95 pH, 30% DO. Viable cell numbers and viability were determined using a ViCell Cell Viability Analyser (Beckman Coulter) and antibody titres were determined using an Immunochemistry System (Beckman Coulter).

## Reproducibility

A hydrolysate containing complex media fed batch process was run in each of the 24 bioreactors using the same model CHO cell line. Viable cell numbers (VCC), viability and titre were measured in each bioreactor and the coefficients of variation (CV) were calculated for each time point. This data was also used to calculate the specific productivity (SPR) for each bioreactor. At each time point CVs were generally less than 10% for each parameter measured and there was no significant difference between different rows of the cassette.

## Cell Line Selection

To demonstrate the potential of this system to identify candidate mAb producing cell lines a series of experiments was carried out using the Micro-24 Bioreactor system and the results compared to those obtained in our standard cell line selection process. After initial screening in static multiwell plates during scale up a further screen in the Duetz Microflask system indicated significant differences in the performance of the remaining cell lines. In the standard hydrolysate containing batch process peak titres varied by up to 60% and there was a 2 fold difference in the overall SPR across the different cell lines.

**Figure 1:**
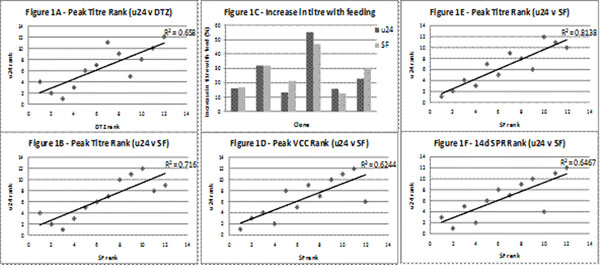
Comparison of performance rank order of clones in different bioreactor systems in a hydrolysate containing medium batch process (Figure 1A and B) and fed batch process (Figure 1C, 1D, 1E and 1F).

Based on titre and SPR data from the Duetz Microflask screen 12 of these cell lines were selected for further evaluation. Using the standard batch process conditions these cell lines were grown in parallel in the Micro-24 Bioreactors, Duetz Microflasks and conventional shake flasks. Although there were some differences in absolute titres the rank order of cell lines was similar in each of the systems tested here (Figure 1a and b) with R^2^=0.66 (Micro-24 v Duetz) and R^2^=0.72 (Micro-24 v shake flasks).The rank order of peak VCC was also similar in the Micro-24 and shake flasks (R^2^ = 0.7) although there was less similarity in overall SPR (R^2^ = 0.4).

This data from the batch process was used to select 6 cell lines for evaluation in the standard fed batch process which was run in parallel in the Micro-24 Bioreactor and shake flasks.

For each cell line the effect of feeding was similar in Micro-24 Bioreactors to shake flasks (Figure 1C) and the rank orders of titre, VCC and SPR achieved in the Micro-24 Bioreactors were similar to those achieved in shake flasks (Figure 1D, E and F).

## Bioreactor validation

Clones ranked 1, 2, 5 and 12 in the Micro-24 Bioreactors were tested in conventional 2 litre bioreactors. In bioreactors the titre produced by the top ranked clone was approximately 20% higher than the clone ranked 12. The remaining 2 clones (ranked 2 and 5 in the Micro-24) produced intermediate titres.

## Product quality

SEC, CE-IEF and NGHC data for the mAb produced in the Micro-24 showed no significant differences to that produced in control shake flasks.

## Process optimization

The Micro-24 Bioreactor also enables early stage process optimisation to be carried out at a small scale. The potential for media development is shown by the data in Table 1A which demonstrates that the same trend in peak titres is observed when cells are grown in 4 different media in shake flasks, conventional bioreactors and the Micro-24. Similarly a further experiment in the Micro-24 Bioreactor to investigate the effect of pH and temperature stepdown on performance demonstrated that condition dependent titre improvements could be identified using this instrument (Table 1B).

**Table 1:** Process optimization.

Table 1A – Medium Development			
**Medium**	**Shake flasks (120mL)**	**Bioreactor (2000mL)**	**Micro-24 (7mL)**

**Medium 1**	100%	100%	100%
**Medium 2**	129%	125%	124%
**Medium 3**	121%	131%	140%
**Medium 4**	153%	175%	159%

**Table 1B – Condition Optimisation**			

	**pH**	**Temperature**	**Relative titre**

**Condition 1**	High	Low	58%
**Condition 2**	High	High	100%
**Condition 3**	Low	Low	96%
**Condition 4**	Low	High	141%

## Discussion

This data demonstrates that the Micro-24 Bioreactor can be used successfully to select high producing cell lines and to carry out initial process optimisation experiments. Although there were some differences in absolute data the rank orders and process trends identified in the Micro-24 Bioreactors were similar to those in conventional systems. Therefore this work has shown that the Micro-24 Bioreactor system could be used to replace shake flasks and bioreactors in cell line evaluation and early process improvement work.

